# Determinants of Stress and Obstacles to Diabetes‐Related Quality of Life in Adults With Type 2 Diabetes: A Cross‐Sectional Interaction–Mediation Analysis

**DOI:** 10.1111/wvn.70168

**Published:** 2026-07-26

**Authors:** Siti Fadlilah, Santi Damayanti, Maria Dyah Kurniasari, Shih Hsin Hsiao, Lee Kai Ling, Po Hao Feng, Hazel Novela Villagracia, Hsiu Ting Tsai

**Affiliations:** ^1^ School of Nursing, College of Nursing Taipei Medical University Taipei Taiwan; ^2^ Program Study of Nursing Universitas Respati Yogyakarta Yogyakarta Indonesia; ^3^ Undergraduate Nursing Program, Faculty of Health Sciences Universitas Kristen Satya Wacana Salatiga Indonesia; ^4^ School of Respiratory Therapy, College of Medicine Taipei Medical University Taipei Taiwan; ^5^ Division of Pulmonary Medicine, Department of Internal Medicine Taipei Medical University Hospital Taipei Taiwan; ^6^ Division of Pulmonary Medicine, Department of Internal Medicine Shuang‐Ho Hospital, Taipei Medical University Taipei Taiwan; ^7^ Medical Surgical Department, College of Nursing University of Ha'il Ha'il Saudi Arabia; ^8^ Center of Excellence for Enhancing Well‐Being in Vulnerable and Chronic Illness Populations, Faculty of Nursing Chulalongkorn University Bangkok Thailand; ^9^ Post‐Baccalaureate Program in Nursing, College of Nursing Taipei Medical University Taipei Taiwan

**Keywords:** diabetes mellitus, fasting blood glucose, psychological stress, quality of life

## Abstract

**Background:**

Diabetes‐related quality of life (DR‐QoL) is shaped by interactions between cardiometabolic dysfunction and psychological stress, yet these mechanisms are rarely examined within a unified framework.

**Aims:**

To evaluate the independent, interactive, and mediating effects of cardiometabolic factors and stress on DR‐QoL obstacles.

**Methods:**

A cross‐sectional study was conducted among 1815 adults with type 2 diabetes from 15 Indonesian provinces (2023–2024). Stress and DR‐QoL obstacles were assessed using validated instruments. Independent and synergistic effects were examined using logistic regression, and mediation was tested via path analysis.

**Results:**

Overweight, obesity, stage 2 systolic hypertension, and abnormal fasting blood glucose (FBG) significantly predicted high stress (AOR = 1.982–20.889; *p* < 0.001), with synergistic interactions markedly amplifying the risk (AOR up to 53.972). DR‐QoL obstacles were strongly associated with cardiometabolic abnormalities (AOR = 1.352–18.420) and high stress (AOR = 18.930), with the highest risk observed in participants with multiple concurrent abnormalities (AOR = 52.097). Mediation analysis identified FBG and stress as key mediators, with stress showing the strongest direct effect on DR‐QoL obstacles (*β* = 0.397).

**Linking Evidence to Action:**

These findings highlight FBG and stress as central mechanistic targets for interventions aimed at reducing DR‐QoL obstacles.

## 1 Introduction

Diabetes mellitus (DM) continues to rise globally, affecting an estimated 589 million adults in 2025 and projected to reach 853 million by 2050 (International Diabetes Federation 2025). Beyond its metabolic consequences, DM carries significant psychological and social burdens that negatively affect mental health, daily functioning, and overall quality of life (QoL). Diabetes‐related stress is a major psychosocial factor that shapes self‐management, glycemic control, and long‐term outcomes (Wojujutari and Sunday 2025).

Fasting blood glucose (FBG), a key marker of glycemic control, is strongly linked to complications; poor FBG regulation contributes to greater symptom burden, reduced autonomy, and lower QoL (Oyewole et al. 2024). Psychological stress further disrupts glycemic control by increasing cortisol release and promoting insulin resistance (Buckert et al. 2024). These bidirectional interactions reflect the bio‐psycho‐metabolic framework, which emphasizes the intertwined metabolic and psychological processes driving overall disease burden.

Diabetes‐related quality of life (DR‐QoL) reflects the physical, emotional, and social challenges of living with diabetes. Patients commonly encounter obstacles, including treatment complexity, fear of complications, social limitations, and gaps in disease knowledge (Pilv et al. 2021). However, evidence on how metabolic and psychological factors jointly shape these obstacles remains limited. Most previous studies examined glycemic control, psychological distress, and QoL separately, often using simple analytical models and relatively small samples (Abualhamael et al. 2023; Albai et al. 2024; Aliche and Idemudia 2024). No research to date has evaluated whether FBG or stress acts as a mediator between metabolic risk and QoL. The broader literature also tends to treat physiological and psychological factors independently, without exploring their interaction in influencing DR‐QoL (Fu et al. 2025; Jafari et al. 2024). Consequently, almost no research has integrated major cardiometabolic indicators and stress within a single analytical framework to assess their direct, combined, and indirect effects on DR‐QoL obstacles.

This gap is essential considering that the combination of cardiometabolic dysfunction and psychological distress has been shown to produce a disease burden that is much greater than the effects of each factor individually. Therefore, this study aims to investigate: (1) the independent and synergistic effects of cardiometabolic factors on stress, (2) the independent and synergistic effects of cardiometabolic and stress on obstacles to DR‐QoL, and (3) the mediating role of the factors in this relationship.

## 2 Methods

### 2.1 Study Design

This study used a cross‐sectional correlational design, from July 2023 to September 2024 at public health centers (PHCs) across 15 provinces in Indonesia.

### 2.2 Participants

A total of 1815 adults with type 2 diabetes were ultimately recruited through consecutive sampling. Eligible participants were≥ 18 years old, had a confirmed diagnosis for at > 1 year, and provided written informed consent. Individuals who were hospitalized, cognitively impaired, experiencing acute psychiatric symptoms, or living with chronic kidney disease, heart failure, or stroke were excluded.

### 2.3 Instruments

#### 2.3.1 Sociodemographic Characteristics

Sociodemographic data were collected using a paper‐based self‐report questionnaire.

#### 2.3.2 Cardiometabolic Assessments

Body mass index (BMI, kg/m^2^) was classified as underweight (< 18.5), normal (18.5–24.9), overweight (25.0–29.9), or obese (≥ 30.0). Blood pressure (BP, mmHg) was recorded as systolic (SBP) and diastolic (DBP) and categorized as non‐hypertension (< 130/80), stage 1 hypertension (130–139/80–89), or stage 2 hypertension (≥ 140/90). FBG (mg/dL) was measured from venous samples after at least 8 h of fasting and categorized as normal (< 126) or abnormal (≥ 126).

#### 2.3.3 Stress

Stress was measured using the Indonesian version of the Problem Areas in Diabetes scale (PAID‐20), previously validated in our study of 1044 participants. The 20‐item scale comprises three domains: emotional and self‐management distress, treatment and social support distress, and complication‐related distress, and showed good reliability (α = 0.858) (Fadlilah et al. 2026). Items are scored on a 0–5 scale, with total scores ranging from 0 to 80 converted to a 0–100 range and categorized as low (0–16), moderate (17–39), or high stress (40–100) (de Wit et al. 2022), with a PAID score of ≥ 40 indicating clinically significant diabetes‐related distress.

#### 2.3.4 Obstacles to DR‐QoL

Obstacles to DR‐QoL were assessed using the Indonesian Diabetes Obstacles Questionnaire (DOQ‐30). Our previous validation demonstrated excellent reliability (α = 0.930) (Fadlilah et al. 2025). The DOQ‐30 contains nine domains and 30 items rated on a 0–4 Likert scale, with scores standardized from 0 (no obstacles) to 1 (worst possible obstacles). Based on the mean total score, participants were classified as having “no obstacle” (< 0.5) or “with obstacles” (≥ 0.5) (Pilv et al. 2021). The two‐item insulin‐use domain was omitted as most participants were not insulin users.

### 2.4 Statistical Analysis

Data were analyzed using SPSS 27 and SmartPLS 4, with significance set at *p* < 0.05. Continuous variables were summarized as mean ± SD and categorical variables as *n* (%). Bivariate associations were examined using Chi‐square or Fisher's exact tests. Synergistic effects were examined by grouping participants according to combinations of abnormal predictors using multinomial and multivariable logistic regression with adjustment for confounders. Synergistic effects on DR‐QoL obstacles were assessed by grouping participants into six exposure categories according to the number of abnormal predictors and modeling these associations with multivariable logistic regression. Mediation pathways were tested using Partial Least Squares‐Structural Equation Modeling with 5000 bootstrap samples, and model fit was evaluated using the standardized root mean square residual (SRMR < 0.08).

### 2.5 Ethical Considerations

This study followed the Declaration of Helsinki and all applicable international ethical standards. Ethical approval was granted by the Ethics Research Committee of Universitas Respati Yogyakarta (090.3/FIKES/PL/V/2023) and the Taipei Medical University Joint Institutional Review Board (N202308068) for the first year. The protocol was renewed for the second year by Ethics Research Committee of Universitas Respati Yogyakarta (090.3/FIKES/PL/VII/2024). All participants were informed about the study procedures and provided written consent before taking part.

## 3 Results

### 3.1 Participant Characteristics

Of the 1815 participants, most were middle‐aged, female, Javanese, employed, and lived in western Indonesia. The majority had primary‐secondary education, low income, national health insurance, were diagnosed at PHCs, had no family history of diabetes, had lived with diabetes for 1–5 years, and reported good medication adherence. Overall, 579 participants experienced high stress, and 1171 reported DR‐QoL obstacles. Findings showed that nearly all sociodemographic and clinical factors (age, education, employment, ethnicity, residence, duration of DM diagnosis, diagnosis site, insurance, medication adherence, and drugs) were significantly associated with both outcomes, while income was related only to DR‐QoL obstacles (Table S1).

### 3.2 Stress and Obstacles to DR‐QoL Profiles

On the PAID‐20, *complication distress* was the most prominent domain (35.82 ± 22.51; 44.5% high stress) and the most troubling item was *worrying about the future and the possibility of serious complications* (Table S2; Figure S1). The highest obstacle was the item “*I do not feel I am being prescribed medication that is right for me*” and the medication domain (0.65 ± 0.268; 80.1%) (Table S3; Figure S2).

### 3.3 Determinants of Stress and Obstacles to DR‐QoL

Participants residing in the middle region and those of Javanese ethnicity showed markedly higher risks for both moderate and high stress. Stress was also more common among individuals who were employed, had lived with diabetes for 6–10 years, and were non‐adherent to medication. For DR‐QoL obstacles, key associated factors included residence in the middle region, diabetes duration of more than 6 years, alcohol use, and medication non‐adherence (Table S4).

Cardiometabolic factors were strong predictors of both stress and DR‐QoL obstacles. Overweight/obesity and hypertension, particularly stage 2, were consistently associated with elevated stress, while abnormal FBG emerged as the most dominant predictor across outcomes. Interaction analyses demonstrated a clear cumulative pattern: combinations of cardiometabolic abnormalities substantially amplified risk, even among individuals with normal BMI, with the highest risk observed when multiple abnormalities co‐occurred. Stress further exacerbated these effects, with moderate and high stress markedly increasing the likelihood of DR‐QoL obstacles, peaking when cardiometabolic dysfunction and stress were simultaneously present (Table 1; detailed estimates in Tables S5 and S6).

**TABLE 1:** Key interaction effects of determinants on stress and DR‐QoL obstacles.

A. Specific cardiometabolic interactions predicting high stress	
Interaction pattern	AOR
BMI‐FBG normal, BP abnormal	2.690
BMI‐BP normal, FBG abnormal	21.049
BMI normal, BP‐FBG abnormal	45.419
BMI abnormal, BP‐FBG normal	1.042
BMI‐BP abnormal, FBG normal	3.446
BMI‐FBG abnormal, BP normal	45.248
All abnormal	53.972

B. Cumulative burden of abnormal determinants on DR‐QoL obstacles	
Number of abnormal determinants	AOR
One abnormality	2.502
Two abnormalities	2.927
Three abnormalities	7.301
Four abnormalities	33.351
All abnormal	52.097

*Note:* Adjusted odds ratios derived from multivariable logistic regression models adjusted for relevant sociodemographic and clinical covariates. Detailed estimates are provided in Tables S5–S6.

**FIGURE 1:**
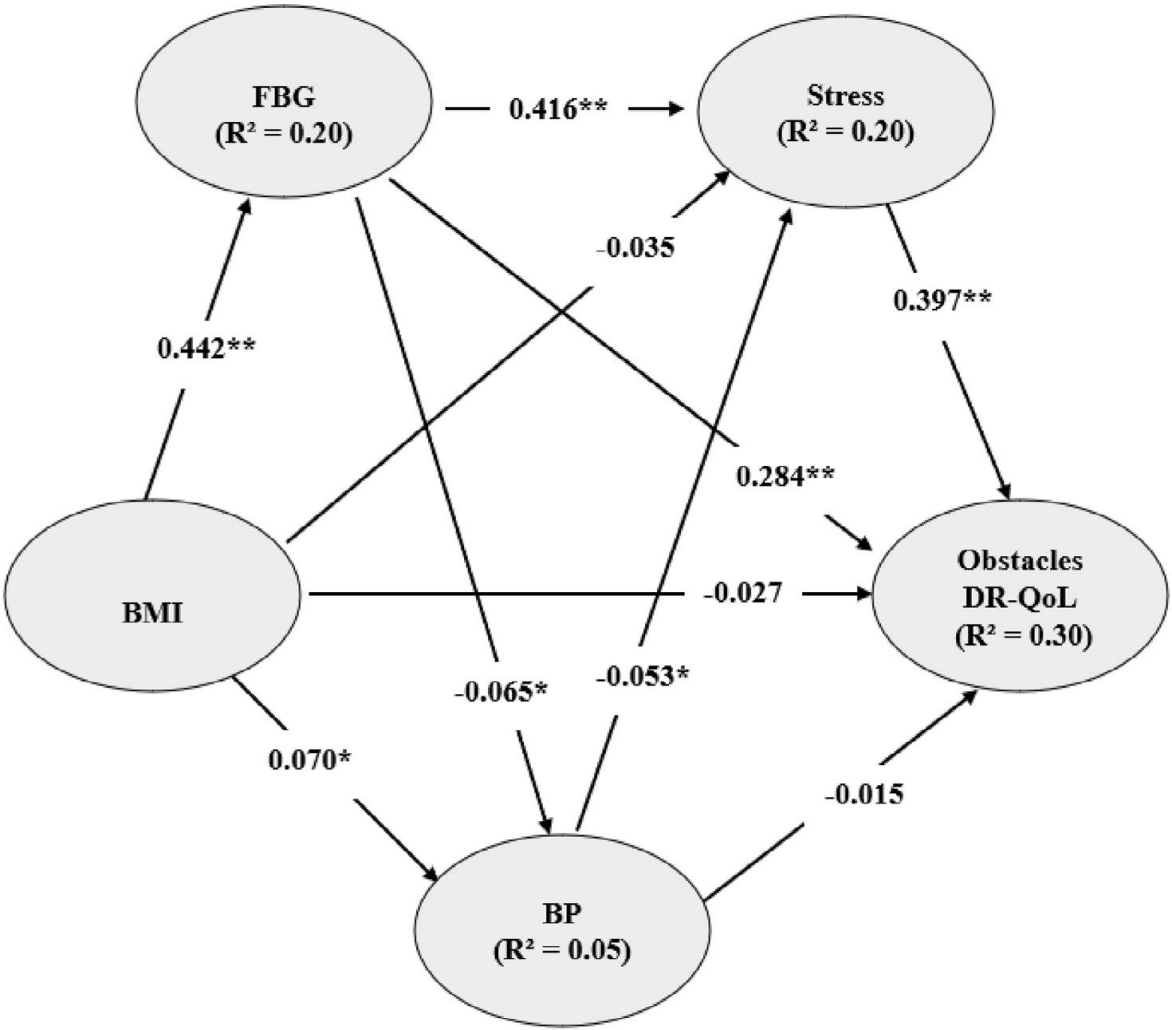
Structural Model of Determinants of Obstacles to DR‐QoL. Standardized coefficients (β) are shown on each path (*p < 0.05; **p < 0.001). R^2^ values indicate the proportion of variance explained in each endogenous construct.

Path analysis (Figure 1; Table S7) indicated that FBG and stress were the strongest predictors of DR‐QoL obstacles. FBG demonstrated significant direct effects on obstacles (*β* = 0.284, *p* < 0.001) and on stress (*β* = 0.416, *p* < 0.001), as well as an indirect effect on obstacles mediated by stress (*β* = 0.165, *p* < 0.001). Stress functioned as the central mediator and exerted the most significant direct effect on obstacles (*β* = 0.397, *p* < 0.001). Neither BMI nor BP showed direct effects on obstacles; however, both displayed small indirect pathways, BMI through FBG and stress (*β* = 0.183, *p* < 0.001) and BP through stress (*β* = −0.021, *p* = 0.011). In terms of total effects, FBG (*β* = 0.451, *p* < 0.001) and stress (*β* = 0.397, *p* < 0.001) remained the most influential predictors. The model accounted for 20% of the variance in stress, 20% in FBG, and 30% in DR‐QoL obstacles, with acceptable fit indices (SRMR = 0.070; NFI = 0.932).

## 4 Discussion

To the best of our knowledge, this is the first study to investigate how cardiometabolic factors interact with stress and operate through mediating pathways to influence obstacles to DR‐QoL in a large primary care population. Three main findings emerged. First, cardiometabolic abnormalities were independently and jointly associated with higher stress, indicating that metabolic dysregulation may contribute to poorer emotional well‐being. Second, the combined presence of cardiometabolic risk and stress exerted a stronger impact on DR‐QoL obstacles than either factor alone, suggesting a cumulative burden on QoL. Third, mediation analysis showed that these relationships primarily operate through indirect pathways, with FBG and stress acting as significant mediators.

These findings align with prior evidence that adiposity affects QoL primarily through biological processes, such as chronic inflammation, insulin resistance, and increased risk of complications, rather than direct functional limitations (Slagter et al. 2015). Although BMI was not directly associated with DR‐QoL obstacles, its indirect effects via FBG and stress highlight interconnected metabolic and psychological pathways. Hypertension showed a similar pattern. Consistent with previous studies, comorbid hypertension is associated with poorer QoL (Shah et al. 2023), likely through worsening endothelial dysfunction and cardiovascular risk (Durante et al. 2024). In our model, BP influenced DR‐QoL obstacles indirectly through stress, reinforcing evidence that cardiovascular burden contributes to QoL impairment partly by increasing emotional distress (Shah et al. 2023).

FBG emerged as the strongest predictor of DR‐QoL obstacles, supporting the evidence that metabolic dysregulation is a significant contributor to disease burden and reduced QoL. Chronic hyperglycemia is associated with complications such as neuropathy, fatigue, visual impairment, and functional decline, which limit daily activities (Jafari et al. 2024; Oyewole et al. 2024). Elevated FBG frequently co‐occurs with higher stress levels, reflecting the bidirectional relationship between glycemic control and psychological distress (Hackett and Steptoe 2017). Stress activates the hypothalamic–pituitary–adrenal axis, increasing cortisol and catecholamine release, which worsens glucose regulation and insulin sensitivity (Fang et al. 2024). Conversely, glucose fluctuations can impair cognition and mood, further intensifying emotional distress (Mascarenhas et al. 2023).

Stress was strongly associated with DR‐QoL obstacles and functioned as a key psychosocial mediator. Psychological distress is known to reduce treatment adherence, weaken self‐care behaviors, and diminish overall QoL (Aliche and Idemudia 2024; Fisher et al. 2010). Poor glycemic control and metabolic complications may further intensify stress, creating a reinforcing cycle between psychological and metabolic vulnerability (Abualhamael et al. 2023; Hackett and Steptoe 2017). Our mediation findings suggest that FBG primarily acts as a physiological mediator of metabolic dysfunction, whereas stress serves as a psychosocial mediator that undermines coping and heightens perceived disease burden (Morales‐Brown et al. 2024).

The combined effects of cardiometabolic abnormalities and elevated stress suggest that DR‐QoL obstacles arise from the accumulated impact of physiological and psychological burdens rather than either domain alone. This synergistic pattern is consistent with evidence that multimorbidity and interacting risk factors accelerate QoL decline (Shah et al. 2023; Teli et al. 2023) and with broader bio‐psycho‐metabolic frameworks emphasizing the interplay of metabolic dysregulation, emotional distress, and psychosocial adaptation (Albai et al. 2024; Aliche and Idemudia 2024; Jafari et al. 2024). Within this complex context, an R^2^ of 0.30 represents a reasonable and expected level of explained variance.

This study found that 31.9% of participants experienced high distress, lower than previously reported (Fisher et al. 2010; Schmitt et al. 2016). Complication‐related distress predominated, a pattern observed in high‐risk settings in developing countries (Berhe et al. 2025). DR‐QoL obstacles were highly prevalent, particularly in treatment‐related domains, consistent with evidence that treatment burden strongly affects QoL (Nicolucci et al. 2013). These findings also align with global evidence that older age, lower education, longer diabetes duration, and comorbidities increase vulnerability to stress and poorer QoL, consistent with social determinants of health frameworks (Hill‐Briggs et al. 2020).

### 4.1 Implications for Nursing Practice

The findings reinforce that diabetes care in primary settings must extend beyond glucose control alone. A risk‐based, integrated model that considers cardiometabolic, psychological, and social factors is essential, as higher obstacle levels are strongly linked to poorer QoL. Priority areas include weight and BMI management, improved glycemic regulation, and routine screening and treatment of stress, consistent with international recommendations that support therapeutic education, glucose monitoring, and mental health care. Because stress and hyperglycemia reinforce each other, regular assessment of distress and coping capacity is crucial. Treatment‐related obstacles further underscore the importance of clear communication, effective management of medication side effects, and simplification of regimens to promote adherence.

Nurses play a central role in delivering integrated care. They conduct early screening for distress and complications, provide tailored education, and support daily self‐management, approaches shown to reduce stress, improve glycemic control, and enhance QoL (Silva et al. 2023; Sun et al. 2025). Their long‐term patient contact enables continuity and emotional support, while collaboration with physicians, nutritionists, and mental‐health professionals ensures that biomedical and psychosocial needs are addressed simultaneously (Dailah 2024). Strengthening the nursing role within integrated care models is therefore critical to interrupting the cycle of stress, hyperglycemia, and declining QoL.

### 4.2 Linking Evidence to Action

This study suggests that cardiometabolic abnormalities and psychological stress jointly contribute to DR‐QoL impairment. These findings emphasize that diabetes management cannot focus solely on metabolic control; it must also consider physical and psychological aspects in an integrated manner. This evidence can be translated into practice through the following steps:

- First, routine assessment of glycemic status and diabetes‐related stress levels needs to be integrated into primary care visits. Early identification of patients with high FBG levels and increased stress allows earlier intervention before QoL worsens.
- Second, diabetes management strategies should simultaneously target metabolic control and psychological well‐being. Programs that combine glucose monitoring, lifestyle counseling, and stress management support can offer more tangible benefits than interventions that focus solely on one aspect.
- Third, patients with multiple cardiometabolic risk factors require closer monitoring and individualized treatment plans. The accumulation of risk factors can significantly impair daily functioning and QoL, requiring a proactive and coordinated approach.
- Fourth, clear communication and shared decision‐making between health workers and patients need to be prioritized in determining therapy plans. Discussion of drug effectiveness, side effects, and treatment burden can help reduce perceived obstacles to therapy and increase adherence.
- Fifth, nurse‐led self‐management education and support programs need to be strengthened. Nurses have an important role in continuous monitoring, patient empowerment, and coordination of care across professions, which can help break the cycle between stress, hyperglycemia, and reduced QoL.

### 4.3 Study Limitations

This study benefits from a large sample, validated instruments, and the use of interaction and mediation analyses that clarify mechanisms relevant to integrated diabetes care. However, its cross‐sectional design limits causal inference, and self‐reported data may introduce recall or social desirability bias. The geographic and cultural context may restrict generalizability, and single‐time‐point FBG measurement may not reflect long‐term glycemic control. Longitudinal studies are needed to further elucidate these relationships.

## 5 Conclusions

This study suggests that complex interactions between cardiometabolic factors and psychological stress may influence DR‐QoL. FBG and stress emerged as significant mediators linking the impact of BMI and BP on DR‐QoL obstacles, highlighting the importance of treatment approaches that consider bio‐psycho‐metabolic mechanisms in an integrated manner.

## Funding

This study was funded by The National Science and Technology Council (NSTC), Taiwan (Grant No. 113‐2314‐B‐038‐055‐MY3).

## Ethics Statement

This study followed the Declaration of Helsinki and all applicable international ethical standards. Ethical approval was granted by the Ethics Research Committee of Universitas Respati Yogyakarta (090.3/FIKES/PL/V/2023) and the Taipei Medical University Joint Institutional Review Board (N202308068) for the first year. The protocol was renewed for the second year by the Ethics Research Committee of Universitas Respati Yogyakarta (090.3/FIKES/PL/VII/2024). All participants were informed about the study procedures and provided written consent before taking part.

## Conflicts of Interest

The authors declare no conflicts of interest.

## Supporting information


**Table S1:** Demographic characteristic of respondents with different level of stress and obstacles diabetes‐related quality of life among people with type 2 diabetes (*N* = 1815).
**Table S2:** Level of stress among the study participants—findings of Problem Areas in Diabetes scale‐20 items (N = 1815).
**Figure S1:** Stress assessment results among participants with diabetes.
**Table S3:** Obstacles for diabetes‐related quality of life among the study participants—findings of Diabetes Obstacles Questionnaire‐28 items (N = 1815).
**Figure S2:** Obstacles to DR‐QoL based on the DOQ‐28 questionnaire.
**Table S4:** Logistic regression analysis of sociodemographic factors with stress and obstacles to DR‐QoL (N = 1815).
**Table S5:** Independent and interaction effects of cardiometabolic factors on stress (N = 1815).
**Table S6:** Independent and interaction effects of cardiometabolic factors and stress on obstacles to DR‐QoL (N = 1815).
**Table S7:** Summary of Path Analysis (N = 1815).

## Data Availability

The data that support the findings of this study are available on request from the corresponding author. The data are not publicly available due to privacy or ethical restrictions.
