# Supplementary material for: Determinants of Stress and Obstacles to Diabetes‐Related Quality of Life in Adults With Type 2 Diabetes: A Cross‐Sectional Interaction–Mediation Analysis
Source: Worldviews Evid Based Nurs. 2026 Jul 26;23(4):e70168. doi: 10.1111/wvn.70168 (PMC13402761; doi:10.1111/wvn.70168)
Supplement: Supplementary file 1 — Table S1: Demographic characteristic of respondents with different level of stress and obstacles diabetes‐related quality of life among people with type 2 diabetes (N = 1815). Table S2: Level of stress among the study participants—findings of Problem Areas in Diabetes scale‐20 items (N = 1815). Figure S1: Stress assessment results among participants with diabetes. Table S3: Obstacles for diabetes‐related quality of life among the study participants—findings of Diabetes Obstacles Questionnaire‐28 items (N = 1815). Figure S2: Obstacles to DR‐QoL based on the DOQ‐28 questionnaire. Table S4: Logistic regression analysis of sociodemographic factors with stress and obstacles to DR‐QoL (N = 1815). Table S5: Independent and interaction effects of cardiometabolic factors on stress (N = 1815). Table S6: Independent and interaction effects of cardiometabolic factors and stress on obstacles to DR‐QoL (N = 1815). Table S7: Summary of Path Analysis (N = 1815). [file WVN-23-0-s001.docx]

**Supplementary files**

**Title: Determinants of Stress and Obstacles to Diabetes-Related Quality of Life in Adults with Type 2 Diabetes: An Interaction–Mediation Analysis**

**Supplementary File 1**

**Table S1. Demographic characteristic of respondents with different level of stress and obstacles diabetes-related quality of life among people with type 2 diabetes (N=1815)–Page 2**

**Supplementary File 2**

**Table S2. Level of stress among the study participants - findings of Problem Areas in Diabetes scale-20 items (N = 1815)–Page 5**

**Supplementary File 3**

**Figure S1. Stress assessment results among participants with diabetes–Page 6**

**Supplementary File 4**

**Table S3. Obstacles for diabetes-related quality of life among the study participants - findings of Diabetes Obstacles Questionnaire-28 items (N = 1815)–Page 7**

**Supplementary File 5**

**Figure S2. Obstacles to DR-QoL based on the DOQ-28 questionnaire–Page 8**

**Supplementary File 6**

**Table S4. Logistic regression analysis of sociodemographic factors with stress and obstacles to DR-QoL (N=1815)–Page 9**

**Supplementary File 7**

**Table S5. Independent and interaction effects of cardiometabolic factors on stress (N=1815)–Page 10**

**Supplementary File 8**

**Table S6. Independent and interaction effects of cardiometabolic factors and stress on obstacles to DR-QoL (N=1815)–Page 11**

**Supplementary File 9**

**Table S7. Summary of Path Analysis (N=1815)–Page 12**

**Supplementary File 1**

**Table S1. Demographic characteristic of respondents with different level of stress and obstacles diabetes-related quality of life among people with type 2 diabetes (*N*=1815)**

| **Characteristic** | **All**  ***N* (%)** | **Stress** | | | | **Obstacles DR-QoL** | | | |
| --- | --- | --- | --- | --- | --- | --- | --- | --- | --- |
|  |  | **Low (n=574)** | **Moderate (*n*=662)** | **High (n=579)** | ***p*** | **No Obstacle (*n*=644)** | **With Obstacles (*n*=1171)** | ***p*** |  |
|  |  | ***n* (%)** | ***n* (%)** | ***n* (%)** |  | ***n* (%)** | ***n* (%)** |  |  |
| **Age (years)** |  |  |  |  |  |  |  |  |  |
| ≤49 | 427 (23.5) | 106 (24.8) | 158 (37.0) | 163 (38.2) | **< .001^a^** | 158 (37.0) | 269 (63.0) | .136^a^ |  |
| 50–64 | 988 (54.4) | 327 (33.1) | 350 (35.4) | 311 (31.5) |  | 331 (33.5) | 657 (66.5) |  |  |
| >64 | 400 (22.0) | 141 (35.3) | 154 (38.5) | 105 (26.3) |  | 155 (38.8) | 245 (61.3) |  |  |
| **Educational level** |  |  |  |  |  |  |  |  |  |
| None | 351 (19.3) | 129 (36.8) | 154 (43.9) | 68 (19.4) | **< .001^a^** | 145 (41.3) | 206 (58.7) | **.039^a^** |  |
| Elementary school | 684 (37.7) | 227 (33.2) | 250 (36.5) | 207 (30.3) |  | 242 (35.4) | 442 (64.6) |  |  |
| Up to senior high school | 681 (37.5) | 186 (27.3) | 233 (34.2) | 262 (38.5) |  | 220 (32.3) | 461 (67.7) |  |  |
| University degree | 99 (5.5) | 32 (32.3) | 25 (25.3) | 42 (42.4) |  | 37 (37.4) | 62 (62.6) |  |  |
| **Occupation** |  |  |  |  |  |  |  |  |  |
| Unemployed | 237 (13.1) | 88 (37.1) | 77 (32.5) | 72 (30.4) | **< .001^a^** | 77 (32.5) | 160 (67.5) | .236^a^ |  |
| Housewife | 325 (17.9) | 124 (38.2) | 141 (43.4) | 60 (18.5) |  | 152 (46.8) | 173 (53.2) |  |  |
| Retired | 90 (5.0) | 32 (35.6) | 35 (38.9) | 23 (25.6) |  | 35 (38.9) | 55 (61.1) |  |  |
| Actively employed | 1163 (64.1) | 330 (28.4) | 409 (35.2) | 424 (36.5) |  | 380 (32.47) | 783 (67.3) |  |  |
| **Ethnicity** |  |  |  |  |  |  |  |  |  |
| Javanese | 1318 (72.6) | 511 (38.8) | 490 (37.2) | 317 (24.1) | **< .001^a^** | 517 (39.2) | 801 (60.8) | **<.001^b^** |  |
| Non-Javanese | 497 (27.4) | 63 (12.7) | 172 (34.6) | 262 (52.7) |  | 127 (25.6) | 370 (74.4) |  |  |
| **Grouping Area** |  |  |  |  |  |  |  |  |  |
| West | 1360 (74.9) | 530 (39.0) | 508 (37.4) | 322 (23.7) | **< .001^a^** | 519 (38.2) | 841 (61.8) | **< .001^a^** |  |
| Middle | 434 (23.9) | 36 (8.3) | 147 (33.9) | 251 (57.8) |  | 118 (27.2) | 316 (72.8) |  |  |
| East | 21 (1.2) | 8 (38.1) | 7 (33.3) | 6 (28.6) |  | 7 (33.3) | 14 (66.7) |  |  |
| **Gender** |  |  |  |  |  |  |  |  |  |
| Male | 769 (42.4) | 246 (32.0) | 255 (33.2) | 268 (34.9) | **.021^a^** | 258 (33.6) | 511 (66.4) | .150^b^ |  |
| Female | 1046 (57.6) | 328 (31.4) | 407 (38.9) | 311 (29.7) |  | 386 (36.9) | 660 (63.1) |  |  |
| **Duration of DM diagnosis (years)** |  |  |  |  |  |  |  |  |  |
| 1–5 | 984 (54.2) | 343 (34.9) | 346 (35.2) | 295 (30.0) | **< .001^a^** | 408 (41.5) | 576 (58.5) | **< .001^a^** |  |
| 6–10 | 588 (32.4) | 156 (26.5) | 204 (34.7) | 228 (38.8) |  | 165 (28.1) | 423 (71.9) |  |  |
| > 10 | 243 (13.4) | 75 (30.9) | 112 (46.1) | 56 (23.0) |  | 71 (29.2) | 172 (70.8) |  |  |
| **Place Diagnosed by DM** |  |  |  |  |  |  |  |  |  |
| Public health center | 1410 (77.7) | 461 (32.7) | 506 (35.9) | 443 (31.4) | **< .001^a^** | 521 (37.0) | 889 (63.0) | **.009^a^** |  |
| Hospital | 311 (17.1) | 94 (30.2) | 128 (41.2) | 89 (28.6) |  | 102 (32.8) | 209 (67.2) |  |  |
| Others | 94 (5.2) | 19 (20.2) | 28 (29.8) | 47 (50.0) |  | 21 (22.3) | 73 (77.8) |  |  |
| **Family history of DM** |  |  |  |  |  |  |  |  |  |
| No | 941 (51.8) | 284 (30.2) | 324 (34.4) | 333 (35.4) | **.020^a^** | 336 (35.7) | 605 (64.3) | .871^b^ |  |
| Yes | 670 (36.9) | 227 (33.9) | 256 (38.2) | 187 (27.9) |  | 239 (35.7) | 431 (64.3) |  |  |
| Do not know | 204 (11.2) | 63 (30.9) | 82 (40.2) | 59 (28.9) |  | 69 (33.8) | 135 (66.2) |  |  |
| **Married status** |  |  |  |  |  |  |  |  |  |
| No | 26 (1.4) | 12 (46.2) | 8 (30.8) | 6 (23.1) | .324^a^ | 11 (42.3) | 15 (57.7) | .729^a^ |  |
| Widow/Widower | 232 (12.8) | 76 (32.8) | 91 (28.0) | 65 (28.0) |  | 80 (34.5) | 152 (65.5) |  |  |
| Yes | 1557 (85.8) | 486 (31.2) | 563 (36.2) | 508 (32.6) |  | 553 (35.5) | 1004 (64.5) |  |  |
| **Health Insurance** |  |  |  |  |  |  |  |  |  |
| No | 88 (4.8) | 15 (17.0) | 23 (26.1) | 50 (56.8) | **< .001^a^** | 24 (27.3) | 64 (72.7) | **.040^a^** |  |
| NHI | 1630 (89.8) | 516 (31.7) | 602 (36.9) | 512 (31.4) |  | 594 (36.4) | 1036 (63.6) |  |  |
| Private insurance | 97 (5.3) | 43 (44.3) | 37 (38.1) | 17 (17.5) |  | 26 (26.8) | 71 (73.2) |  |  |
| **Income (per month) (IDR)** |  |  |  |  |  |  |  |  |  |
| < 2,500,000 | 956 (52.7) | 323 (33.8) | 351 (36.7) | 282 (29.5) | .761^a^ | 383 (40.1) | 573 (59.9) | **< .001^a^** |  |
| 2,500,000 to 5,000,000 | 709 (39.1) | 203 (28.6) | 261 (36.8) | 245 (34.6) |  | 219 (30.9) | 490 (69.1) |  |  |
| > 5,000,000 | 150 (8.3) | 48 (32.0) | 50 (33.3) | 52 (34.7) |  | 42 (28.0) | 108 (72.0) |  |  |
| **Alcoholic Behaviour** |  |  |  |  |  |  |  |  |  |
| No | 1742 (96.0) | 558 (32.0) | 636 (36.5) | 548 (31.5) | .084^a^ | 630 (36.2) | 1112 (64.5) | **.003^b^** |  |
| Yes | 73 (4.0) | 16 (21.9) | 26 (35.6) | 31 (42.5) |  | 14 (19.2) | 59 (80.8) |  |  |
| **Adherence Diabetic Drug Consumption** | | | | | | | | |  |
| No | 887 (48.9) | 216 (24.4) | 320 (36.9) | 351 (39.6) | **< .001^a^** | 254 (28.6) | 633 (71.4) | **<.001^b^** |  |
| Yes | 928 (51.1) | 358 (38.6) | 342 (36.9) | 228 (24.6) |  | 390 (42.0) | 538 (58.0) |  |  |
| **Drugs** |  |  |  |  |  |  |  |  |  |
| Non-adherence | 887 (48.9) | 216 (24.4) | 320 (36.9) | 351 (39.6) | **< .001^a^** | 254 (28.6) | 633 (71.4) | **< .001^a^** |  |
| Metformin | 564 (31.1) | 186 (33.0) | 231 (41.0) | 147 (26.1) |  | 221 (39.2) | 343 (60.8) |  |  |
| Glimepiride | 92 (5.1) | 60 (65.2) | 16 (17.4) | 16 (17.4) |  | 63 (68.5) | 29 (31.5) |  |  |
| Insulin | 103 (5.7) | 56 (54.4) | 20 (19.4) | 27 (26.2) |  | 54 (52.4) | 49 (47.6) |  |  |
| Metformin + Glimepiride | 96 (5.3) | 27 (28.1) | 47 (49.0) | 22 (22.9) |  | 27 (28.1) | 69 (71.9) |  |  |
| Metformin + Insulin | 44 (2.4) | 15 (34.1) | 20 (45.5) | 9 (20.5) |  | 13 (29.5) | 31 (70.5) |  |  |
| Others | 29 (1.6) | 14 (48.3) | 8 (27.6) | 7 (24.1) |  | 12 (41.4) | 17 (58.6) |  |  |
| **BMI (kg/m^2^)** |  |  |  |  |  |  |  |  |  |
| Normal (18.5–24.9) | 970 (53.4) | 374 (38.6) | 377 (38.9) | 219 (22.6) | **< .001^a^** | 444 (45.8) | 526 (54.2) | **< .001^a^** |  |
| Underweight (< 18.5) | 66 (3.6) | 21 (31.8) | 19 (28.8) | 26 (39.4) |  | 21 (31.8) | 45 (68.2) |  |  |
| Overweight (25.0–29.9) | 611 (33.7) | 132 (2126) | 213 (34.9) | 266 (43.5) |  | 129 (21.1) | 482 (78.9) |  |  |
| Obese (> 29.9) | 168 (9.3) | 47 (28.0) | 53 (31.5) | 68 (40.5) |  | 50 (29.8) | 118 (70.2) |  |  |
| **SBP (**mmHg) |  |  |  |  |  |  |  |  |  |
| Non-hypertension (< 130) | 503 (27.7) | 211 (41.9) | 183 (36.4) | 109 (18.8) | **< .001^a^** | 238 (47.3) | 265 (52.7) | **< .001^a^** |  |
| Hypertension Stage 1 (130–139) | 485 (26.7) | 161 (33.2) | 169 (34.8) | 155 (32.0) |  | 168 (34.6) | 317 (65.4) |  |  |
| Hypertension Stage 2 (≥ 140) | 827 (45.6) | 202 (24.4) | 310 (37.5) | 315 (38.1) |  | 235 (28.8) | 589 (71.2) |  |  |
| **DBP (**mmHg) |  |  |  |  |  |  |  |  |  |
| Non-hypertension (< 80) | 434 (23.9) | 174 (40.1) | 147 (33.9) | 113 (26.0) | **< .001^a^** | 183 (42.2) | 251 (57.8) | **.003^a^** |  |
| Hypertension Stage 1 (80-89) | 781 (43.0) | 274 (35.1) | 275 (35.2) | 232 (29.7) |  | 266 (34.1) | 515 (65.9) |  |  |
| Hypertension Stage 2 (≥ 90) | 600 (33.1) | 126 (21.0) | 240 (40.0) | 234 (39.0) |  | 195 (32.5) | 405 (67.5) |  |  |
| **FBG (gr/dL)** |  |  |  |  |  |  |  |  |  |
| Normal | 745 (41.0) | 419 (56.2) | 249 (33.4) | 77 (10.3) | **< .001^a^** | 521 (69.9) | 224 (30.1) | **<.001^b^** |  |
| High | 1070 (59.0) | 155 (14.5) | 413 (38.6) | 502 (46.9) |  | 123 (11.5) | 947 (88.5) |  |  |

^a^ Chi-squared; ^b^ Fisher exact test. Bold indicates significancy.

BMI, body mass index; DBP, diastolic blood pressure; DM, diabetes mellitus; DR-QoL, diabetes-related quality of life*;* FBG, fasting blood glucose; n, number; NHI, national health insurance*; p*, *p* value; SBP, systolic blood pressure; SD, standard deviation.

| **Table S2. Level of stress among the study participants - findings of Problem Areas in Diabetes scale-20 items (N = 1815)** | | | | |  |
| --- | --- | --- | --- | --- | --- |
| **Subscales and Items** | **Mean ± SD** | **Level of Stress** | | |  |
|  |  | **Low**  ***n* (%)** | **Moderate  *n* (%)** | **High**  ***n* (%)** | |
| ***Emotional and diabetes self-management distress*** | **27.67 ± 18.524** | **617 (34.0)** | **701 (38.6)** | **497 (****27.4)** | |
| Feeling scared when you think about living with diabetes | 1.18 ± 1.155 |  |  |  | |
| Feelings of deprivation regarding food and meals | 1.15 ± 1.170 |  |  |  | |
| Feeling depressed when you think about living with diabetes | 1.17 ± 1.135 |  |  |  | |
| Not knowing if your mood or feelings are related to your diabetes | 1.07 ± 1.080 |  |  |  | |
| Feeling overwhelmed by your diabetes | 1.19 ± 1.123 |  |  |  | |
| Worrying about low blood sugar reactions | 1.24 ± 1.161 |  |  |  | |
| Feeling angry when you think about living with diabetes | 1.04 ± 1.123 |  |  |  | |
| Feeling constantly concerned about food and eating | 1.24 ± 1.129 |  |  |  | |
| Not “accepting” your diabetes | 0.97 ± 1.130 |  |  |  | |
| Feeling that diabetes is taking up too much of your mental and physical energy every day | 1.13 ± 1.149 |  |  |  | |
| Feeling “burned out” by the constant effort needed to manage diabetes | 1.15 ± 1.105 |  |  |  | |
| ***Treatment-related and social support distress*** | **22.91 ± 17.912** | **822 (45.3)** | **639 (35.2)** | **354 (****19.5)** | |
| Not having clear and concrete goals for your diabetes care | 1.05 ± 1.146 |  |  |  | |
| Feeling discouraged with your diabetes treatment plan? | 0.94 ± 1.096 |  |  |  | |
| Uncomfortable in social situations related to your diabetes care (e.g., people telling you what to eat)? | 0.95 ± 1.118 |  |  |  | |
| Feeling unsatisﬁed with your diabetes physician | 0.95 ± 1.142 |  |  |  | |
| Feeling alone with your diabetes | 0.92 ± 1.119 |  |  |  | |
| Feeling that your friends and family are not supportive of your diabetes management efforts | 0.85 ± 1.054 |  |  |  | |
| ***Complications distress*** | **35.82 ± 22.511** | **477 (26.3)** | **531 (29.3)** | **807 (****44.5)** | |
| Worrying about the future and the possibility of serious complications | 1.59 ± 1.238 |  |  |  | |
| Feelings of guilt or anxiety when you get off track with your diabetes management | 1.32 ± 1.153 |  |  |  | |
| Coping with complications of diabetes | 1.45 ± 1.231 |  |  |  | |

**Supplementary File 2**


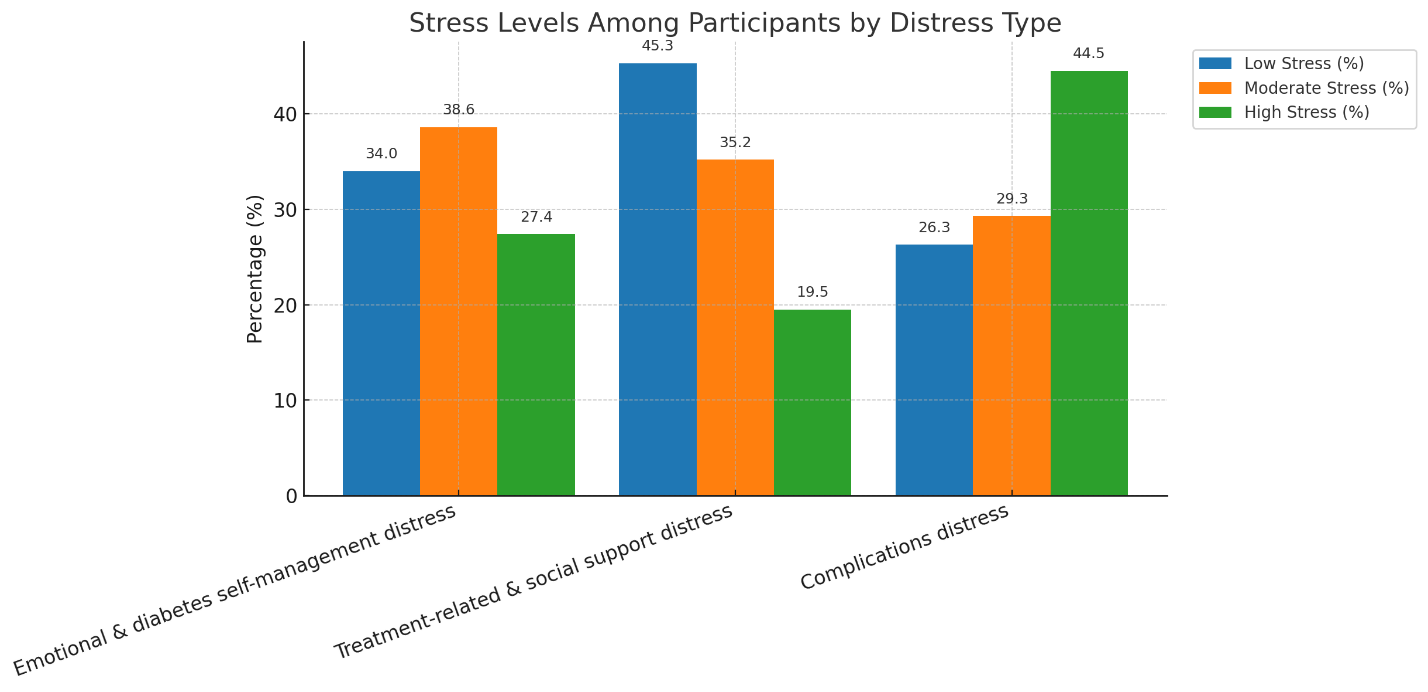
**Supplementary File 3**

**Figure S1. Stress assessment results among participants with diabetes.**

(A). Mean scores (±SD) for the three PAID-20 distress domains, ranked from highest to lowest.

(B). Distribution of stress levels (low, moderate, and high) among participants within each distress domain. Percentages represent the proportion of participants in each category.


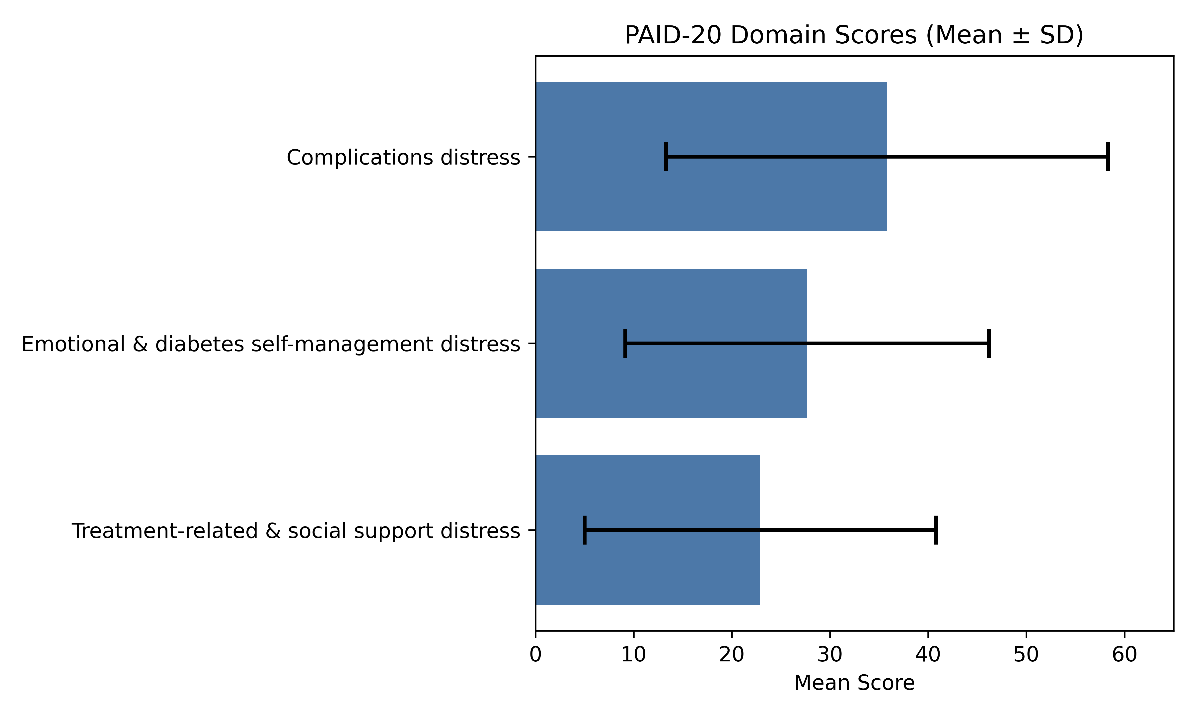
**Supplementary File 4**

**(B)**

**(A)**

**Table S3. Obstacles for diabetes-related quality of life among the study participants - findings of Diabetes Obstacles Questionnaire-28 items (N = 1815)**

| **Subscales and Items** | **Mean ± SD** | **Obstacles to DR-QoL** | |
| --- | --- | --- | --- |
|  |  | **No Obstacle**  ***n* (%)** | **With Obstacles *n* (%)** |
| ***Self-monitoring*** | **0.60 ± 0.215** | **471 (26.0)** | **1344 (74.0)** |
| Self-monitoring makes me feel frustrated | 0.61 ± 0.305 |  |  |
| find it too uncomfortable to self-monitor | 0.62 ± 0.296 |  |  |
| I find it especially hard to test when I am busy | 0.59 ± 0.295 |  |  |
| Self-monitoring makes me fearful of a high reading | 0.60 ± 0.295 |  |  |
| ***Relationships with Medical Professionals*** | **0.62 ± 0.222** | **487 (26.8)** | **1328 (73.2)** |
| I am not assisted in setting realistic targets for changing my lifestyle | 0.65 ± 0.300 |  |  |
| I have not been told what to expect from my treatment | 0.62 ± 0.309 |  |  |
| The good and bad aspects of each choice have not been discussed with me | 0.59 ± 0.327 |  |  |
| Treatment alternatives are not explained to me | 0.63 ± 0.312 |  |  |
| ***Knowledge of the Disease*** | **0.56 ± 0.182** | **573 (31.6)** | **1242 (68.4)** |
| I would manage my diabetes much better if I had encouragement socially | 0.51 ± 0.324 |  |  |
| I have difficulty understanding the information from literature | 0.55 ± 0.281 |  |  |
| I do not know as much as I need to know to manage my diabetes | 0.56 ± 0.280 |  |  |
| I do not know as much as I need to know about the consequences of having diabetes | 0.59 ± 0.273 |  |  |
| I do not know enough about the treatment for diabetes | 0.59 ± 0.284 |  |  |
| ***Support from Others*** | **0.60 ± 0.238** | **491 (27.1)** | **1324 (72.9)** |
| I feel I get little support from my family | 0.57 ± 0.323 |  |  |
| I feel I get little support from my friends | 0.59 ± 0.309 |  |  |
| I feel very alone with my diabetes | 0.65 ± 0.300 |  |  |
| ***Exercising*** | **0.59 ± 0.212** | **486 (26.8)** | **1329 (73.2)** |
| I have not found an exercise I enjoy | 0.60 ± 0.295 |  |  |
| I lack the motivation to exercise | 0.56 ± 0.302 |  |  |
| I am unable to fit exercise into my lifestyle | 0.57 ± 0.300 |  |  |
| I am unable to afford the cost of exercising on a regular basis | 0.61 ± 0.310 |  |  |
| ***Medication*** | **0.65 ± 0.268** | **362 (19.9)** | **1453 (80.1)** |
| I do not feel I am being prescribed a medication dose that is right for me | 0.64 ± 0.312 |  |  |
| I do not feel I am being prescribed medication that is right for me | 0.65 ± 0.314 |  |  |
| ***Lifestyle Changes*** | **0.61 ± 0.224** | **487 (26.8** | **1328 (73.2)** |
| My diabetes has placed a strain on my personal relationships | 0.61 ± 0.300 |  |  |
| Changes in my diet have put a strain on my family | 0.62 ± 0.304 |  |  |
| I feel resentful that I am obliged to change my eating habits | 0.57 ± 0.311 |  |  |
| My diabetic diet spoils my social life | 0.62 ± 0.315 |  |  |
| ***Uncertainty about a consultation*** | **0.62 ± 0.255** | **410 (22.6)** | **1405 (77.4)** |
| I feel a sense of helpless when consulting with nurses | 0.62 ± 0.305 |  |  |
| The way that I was told that I had diabetes made feel afraid | 0.61 ± 0.305 |  |  |


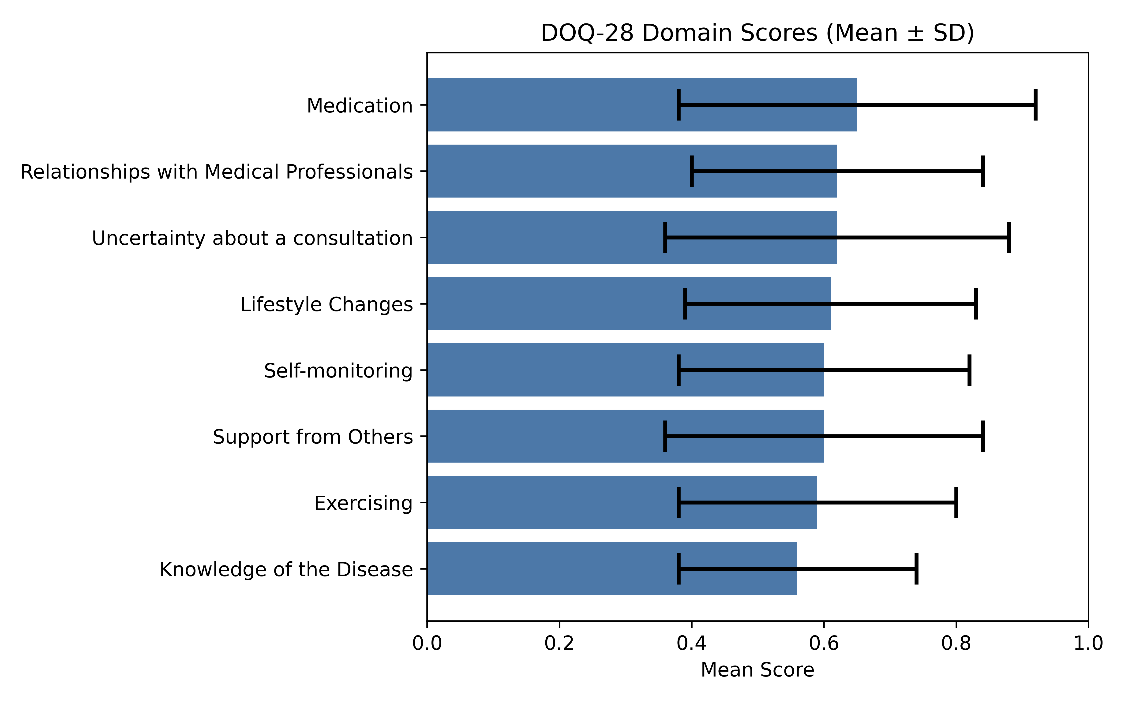

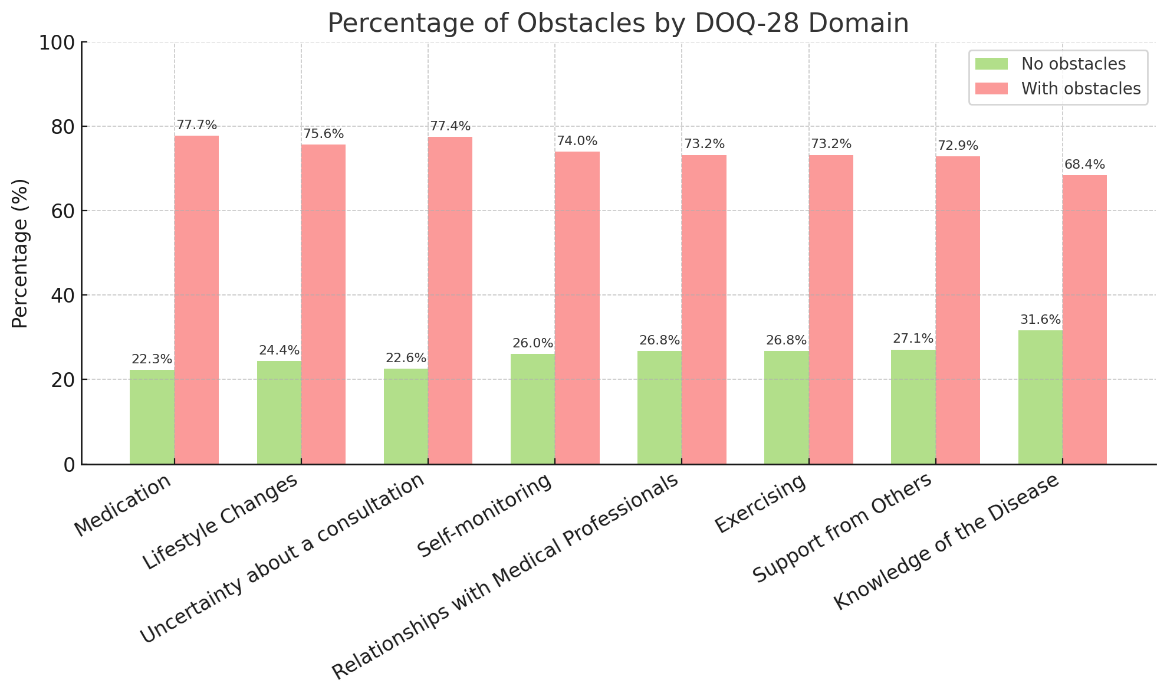
**Supplementary File 5**

**(D)**

**(C)**

**Figure S2. Obstacles to DR-QoL based on the DOQ-28 questionnaire.**

(C). Mean scores (±SD) for each DOQ-28 domain, ranked from highest to lowest, illustrating the relative burden of different obstacle types.

(D). Proportion of participants reporting no obstacles versus experiencing obstacles (scores ≥ 0.5) in each DOQ-28 domain. Percentages represent the proportion of respondents within each category.

**Supplementary File 6**

**Table S4. Logistic regression analysis of sociodemographic factors with stress and obstacles to DR-QoL (*N*=1815)**

| **Characteristic** | **Stress** | | | | | | **Obstacles to DR-QoL** | |
| --- | --- | --- | --- | --- | --- | --- | --- | --- |
|  | **Moderate (*n*=662)** | | **High (n=579)** | | | **With Obstacles (*n*=1171)** | | |
|  | **OR (95% CI)** | ***p^a^*** | **OR (95% CI)** | ***p^a^*** | **OR (95% CI)** | | | ***p^b^*** |
| **Age (years)** |  |  |  |  |  | | |  |
| 50–64 | 0.718 (0.538–0.958) | **.024** | 0.618 (0.463–0.826) | **.001** | 1.166 (0.920–1.477 | | | .204 |
| >64 | 0.733 (0.524–1.025) | .069 | 0.484 (0.341–0.688) | **< .001** | 0.928 (0.701–1.230) | | | .605 |
| **Education** |  |  |  |  |  | | |  |
| None | 1.528 (0.861–2.710) | .147 | 0.402 (0.233–0.693) | **.001** | 0.848 (0.536–1.342) | | | .481 |
| Elementary school | 1.410 (0.811–2.451) | .224 | 0.695 (0.423–1.142) | .151 | 1.090 (0.705–1.686) | | | .699 |
| ≤Senior high school | 1.603 (0.918–2.800) | .097 | 1.073 (0.653–1.764) | .780 | 1.251 (0.807–1.937) | | | .317 |
| **Occupation** |  |  |  |  |  | | |  |
| Housewife | 1.300 (0.880–1.919) | .187 | 0.591 (0.382–0.917) | **.019** | 1.008 (0.748–1.359) | | | .956 |
| Retired | 1.250 (0.708–2.207) | .442 | 0.878 (0.473–1.633) | .682 | 0.552 (0.430–0.709) | | | **< .001** |
| Actively employed | 1.416 (1.010–1.987) | **.044** | 1.570 (1.114–2.213) | **.010** | 0.763 (0.491–1.186) | | | .229 |
| **Ethnicity** (Javanese) | 2.842 (2.076–3.890) | **< .001** | 6.691 (4.914–9.110) | **< .001** | 0.531 (0.422–0.668) | | | **< .001** |
| **Grouping Area** |  |  |  |  |  | | |  |
| Middle | 4.260 (2.901– 6.256) | **< .001** | 11.476 (7.881–16.710) | **< .001** | 1.653 (1.303–2.097) | | | **< .001** |
| East | 0.861 (0.329–2.536) | .861 | 1.234 (0.424–3.590) | .699 | 1.234 (0.495–3.078) | | | .652 |
| **Gender** (Male) | 0.835 (0.665–1.049) | .122 | 1.149 (0.911–1.450) | .242 | 1.158 (0.953–1.408) | | | **< .001** |
| **Duration of DM diagnosis (years)** | | | | | | | | |
| 6–10 | 1.296 (1.003–1.675) | **.047** | 1.699 (1.315–2.196) | **< .001** | 1.816 (1.457–2.263) | | | **< .001** |
| > 10 | 1.480 (1.066–2.056) | **.019** | 0.868 (0.594–1.269) | .465 | 1.716 (1.266–2.326) | | | **< .001** |
| **Place Diagnosed by DM** | | | | | | | | |
| Public health center | 0.745 (0.410–1.352) | .333 | 0.388 (0.224–0.672) | **< .001** | 0.491 (0.299–0.807) | | | **.005** |
| Hospital | 0.924 (0.487–1.753) | .809 | 0.383 (0.209–0.702) | **.002** | 0.589 (0.344–1.011) | | | .055 |
| **Family history of DM** | | | | | | | | |
| Do not know | 1.154 (0.794–1.677) | .452 | 1.137 (0.759–1.703) | .534 | 1.085 (0.780–1.510) | | | .629 |
| No | 1.012 (0.796–1.285) | .952 | 1.423 (1.108–1.828) | **.006** | 0.998 (0.812–1.228) | | | .988 |
| **Married status** |  |  |  |  |  | | |  |
| Yes | 1.738 (0.705–4.286) | .230 | 2.091 (0.778–5.614) | .143 | 1.047 (0.783–1.399) | | | .759 |
| Widow/Widower | 1.796 (0.698–4.621) | .225 | 1.711 (0.608–4.812) | .309 | 0.751 (0.343–1.647) | | | .475 |
| **Health Insurance** |  |  |  |  |  | | |  |
| Private insurance | 1.314 (0.679–2.545) | .418 | 3.359 (1.863–6.059) | **< .001** | 1.529 (0.946–2.471) | | | .083 |
| No | 0.738 (0.468–1.162) | .190 | 0.398 (0.224–0.708) | **.002** | 1.566 (0.988–2.481) | | | .056 |
| **Monthly income (IDR)** | | | | | | | | |
| < 2,500,000 | 1.043 (0.683–1.594) | .845 | 0.806 (0.528–1.231) | .318 | 0.582 (0.398–0.850) | | | **.005** |
| 2,500,000–5,000,000 | 1.234 (0.798–1.910) | .345 | 1.114 (0.722–1.720) | .626 | 0.870 (0.589–1.286) | | | .485 |
| **Alcohol consumption** (Yes) | 1.426 (0.757–2.685) | .272 | 1.973 (1.067–3.648) | **.030** | 2.388 (1.322–4.311) | | | **.004** |
| **Adherence of medication** (No) | 1.551 (1.235–1.947) | **< .001** | 2.552 (2.013–3.235) | **< .001** | 1.807 (1.486–2.196) | | | **< .001** |

^a^ multinomial logistic regression; ^b^ logistic regression. Bold indicates significancy.

CI, confident interval; DM, diabetes mellitus; DR-QoL, diabetes-related quality of life*;* IDR, Indonesian rupiahs; OR; odd ratio; *p*, *p* value.

**Supplementary File 7**

**Table S5.** **Independent and interaction effects of cardiometabolic factors on stress (*N*=1815)**

| **Variable** | **Level of Stress** | | | | | | | |
| --- | --- | --- | --- | --- | --- | --- | --- | --- |
|  | **Moderate** | | | | **High** | | | |
|  | **OR (95% CI)** | ***p*^a^** | **AOR (95% CI)** | ***p*^b^** | **OR (95% CI)** | ***p*^a^** | **AOR (95% CI)** | ***p*^b^** |
| **BMI** |  |  |  |  |  |  |  |  |
| Underweight | 0.898 (0.475–1.697) | .739 | 0.789 (0.404–1.540) | .487 | 2.114 (1.162–3.848) | **.014** | 2.139 (1.086–4.214) | **.028** |
| Overweight | 1.601 (1.234–2.076) | **< .001** | 1.526 (1.160–2.009) | **.003** | 3.441 (2.635–4.495) | **< .001** | 2.966 (2.208–3.984) | **< .001** |
| Obese | 1.119 (0.737–1.699) | .599 | 1.049 (0.671–1.640) | .834 | 2.471 (1.644–3.714) | **< .001** | 1.982 (1.244–3.159) | **.004** |
| **SBP** |  |  |  |  |  |  |  |  |
| Hypertension Stage 1 | 1.210 (0.903–1.622) | .202 | 1.419 (1.035–1.945) | **.030** | 1.864 (1.354–2.565) | **< .001** | 2.378 (1.649–3.428) | **< .001** |
| Hypertension Stage 2 | 1.769 (1.357–2.308) | **< .001** | 1.832 (1.357–2.441) | **< .001** | 3.019 (2.257–4.038) | **< .001** | 3.503 (2.509–4.891) | **< .001** |
| **DBP** |  |  |  |  |  |  |  |  |
| Hypertension Stage 1 | 1.188 (0.901–1.566) | .221 | 1.261 (0.939–1.694) | .123 | 1.304 (0.971–1.750) | .077 | 1.350 (0.967–1.885) | .078 |
| Hypertension Stage 2 | 2.255 (1.657–3.067) | **< .001** | 2.113 (1.522–2.934) | **< .001** | 2.860 (2.075–3.942) | **< .001** | 2.462 (1.714–3.537) | **< .001** |
| **FBG** (High) | 4.484 (3.519–5.713) | **< .001** | 5.097 (3.892–6.675) | **< .001** | 17.624 (13.024–23.848) | **< .001** | 20.889 (14.794–29.494) | **< .001** |
| **BMI–BP–FBG interaction** | | | | | | | | |
| All normal (n=117) |  |  |  |  |  |  |  |  |
| BMI–FBG normal, BP abnormal (n=449) | 1.855 (1.175–2.929) | **.008** | 2.071 (1.263–3.396) | **.004** | 2.515 (1.096–5.772) | **.030** | 2.690 (1.094–6.614) | **.031** |
| BMI–BP normal, FBG abnormal (n=41) | 6.703 (2.704–16.616) | **< .001** | 7.431 (2.871–19.234) | **< .001** | 15.321 (4.639–50.603) | **< .001** | 21.049 (5.723–77.426) | **< .001** |
| BMI normal, BP–FBG abnormal (n=363) | 5.936 (3.577–9.853) | **< .001** | 8.042 (4.630–13.967) | **< .001** | 26.959 (11.779–61.701) | **< .001** | 45.419 (18.330–112.541) | **< .001** |
| BMI abnormal, BP–FBG normal (n=40) | 0.783 (0.333–1.845) | .577 | 0.816 (0.329–2.023) | .661 | 1.194 (0.289–4.938) | .807 | 1.042 (0.223–4.871) | .959 |
| BMI–BP abnormal, FBG normal (n=140) | 1.009 (0.573–1.776) | .976 | 1.164 (0.638–2.124) | .621 | 2.177 (0.858–5.528) | .102 | 3.446 (1.263–9.406) | **.016** |
| BMI–FBG abnormal, BP normal (n=84) | 10.563 (4.590–24.307) | **< .001** | 13.338 (5.460–32.584) | **< .001** | 44.571 (15.384–129.133) | **< .001** | 45.248 (14.124–144.956) | **< .001** |
| All abnormal (n=581) | 6.447 (3.954–10.510) | **< .001** | 7.733 (4.543–13.163) | <.001 | 44.571 (19.766–100.508) | **< .001** | 53.972 (22.188–131.284) | **< .001** |

^a^ unadjusted logistic regression; ^b^ adjusted logistic regression by age, education, occupation, ethnicity, area, gender, duration of DM, placed diagnose, family history, health insurance, adherence of medication, and drugs. Bold indicates significancy.

AOR, adjusted odd ratio; BMI, body mass index; DBP, diastolic blood pressure; FBG, fasting blood glucose; n, number; OR, odd ratio; *p*, *p* value; SBP, systolic blood pressure.

**Supplementary File 8**

**Table S6. Independent and interaction effects of cardiometabolic factors and stress on obstacles to DR-QoL (*N*=1815)**

| **Variable** | **Obstacles DR–QoL** | | | |
| --- | --- | --- | --- | --- |
|  | **OR (95%CI)** | ***p*^a^** | **AOR (95%CI)** | ***p*^b^** |
| **BMI** |  |  |  |  |
| Underweight | 1.809 (1.061–3.083) | **.029** | 1.882 (1.076–3.290) | **.027** |
| Overweight | 3.154 (2.502–3.976) | **< .001** | 3.012 (2.359–3.846) | **< .001** |
| Obese | 1.992 (1.398–2.838) | **< .001** | 1.992 (1.422–3.027) | **< .001** |
| **SBP** |  | **< .001** |  |  |
| Hypertension Stage 1 | 1.695 (1.312–2.189) | **< .001** | 1.564 (1.189–2.058) | **.001** |
| Hypertension Stage 2 | 2.223 (1.764–2.800) | **< .001** | 2.183 (1.706–2.793) | **< .001** |
| **DBP** |  |  |  |  |
| Hypertension Stage 1 | 1.412 (1.109–1.797) | **.005** | 1.352 (1.034–1.725) | **.027** |
| Hypertension Stage 2 | 1.514 (1.172–1.956) | **.001** | 1.438 (1.096–1.887) | **.009** |
| **FBG** (High) | 17.907 (14.022–22.869) | **< .001** | 18.420 (14.137–23.999) | **< .001** |
| **Stress** |  |  |  |  |
| Moderate | 3.826 (3.021–4.845) | **< .001** | 3.742 (2.899–4.831) | **< .001** |
| High | 16.108 (11.708–22.160) | **< .001** | 18.930 (13.179–27.190) | **< .001** |
| **BMI–BP–FBG–Stress interaction** | | | | |
| All normal (n=78) |  |  |  |  |
| One of BMI–SBP–DBP–FBG–Stress abnormal (n=149) | 2.233 (1.221–4.450) | **.022** | 2.502 (1.222–5.123) | **.012** |
| Two of BMI–SBP–DBP–FBG–Stress abnormal (n=306) | 2.612 (1.377–4.956) | **.003** | 2.927 (1.503–5.700) | **.002** |
| Three of BMI–SBP–DBP–FBG–Stress abnormal (n=443) | 7.374 (3.947–13.777) | **< .001** | 7.301 (3.800–14.028) | **< .001** |
| Four of BMI–SBP–DBP–FBG–Stress abnormal (n=437) | 32.034 (16.629–61.710) | **< .001** | 33.351 (16.801–66.207) | **< .001** |
| All abnormal (n=402) | 49.324 (24.869–97.828) | **< .001** | 52.097 (25.332–107.142) | **< .001** |

^a^ unadjusted logistic regression; ^b^ adjusted logistic regression by education, ethnicity, area, duration of DM, first place diagnosed, health insurance, monthly income, alcoholism, adherence of medication, and drugs. Bold indicates significancy.

AOR, adjusted odd ratio; BMI, body mass index; DBP, diastolic blood pressure; DR-QoL, diabetes-related quality of life*;* FBG, fasting blood glucose; n, number; OR, odd ratio; *p*, *p* value; SBP, systolic blood pressure.

| **Supplementary File 9** | | | | | |
| --- | --- | --- | --- | --- | --- |
| **Table S7. Summary of Path Analysis (*N*=1815)** | | | | | |
|  |  |  |  |  | |
| **Path** | ***β*** | **t-stat.** | ***p*** | **Bootstrapping (95% CICB)** | |
|  |  |  |  | **Lower (2.5%)** | **Upper (97.5%)** |
| **Direct Effects** |  |  |  |  |  |
| BMI 🡪 Obstacles on DR-QoL | -0.027 | 1.158 | .247 | -0.073 | 0.019 |
| BMI 🡪 FBG | 0.442 | 22.231 | **< .001** | 0.403 | 0.481 |
| BMI 🡪 Stress | -0.035 | 1.446 | .148 | -0.084 | 0.012 |
| BMI 🡪 SBP | 0.070 | 2.532 | **.011** | 0.015 | 0.123 |
| FBG 🡪 Obstacles on DR-QoL | 0.284 | 10.983 | **< .001** | 0.231 | 0.331 |
| FBG 🡪 Stress | 0.416 | 17.381 | **< .001** | 0.367 | 0.461 |
| FBG 🡪 BP | -0.065 | 2.365 | **.018** | -0.116 | -0.009 |
| Stress 🡪Obstacles on DR-QoL | 0.397 | 19.106 | **< .001** | 0.356 | 0.437 |
| BP 🡪 Obstacles on DR-QoL | -0.015 | 0.752 | .452 | -0.056 | 0.024 |
| BP 🡪 Stress | -0.053 | 2.574 | **.010** | -0.094 | -0.013 |
| **Total Indirect Effects** |  |  |  |  |  |
| BMI 🡪 Obstacles on DR-QoL | 0.183 | 11.091 | **< .001** | 0.150 | 0.215 |
| BMI 🡪 Stress | 0.182 | 13.473 | **< .001** | 0.156 | 0.209 |
| BMI 🡪 BP | -0.029 | 2.346 | **.019** | -0.052 | -0.004 |
| FBG 🡪 Obstacles on DR-QoL | 0.168 | 14.058 | **< .001** | 0.145 | 0.193 |
| FBG 🡪 Stress | 0.003 | 1.683 | .092 | 0.000 | 0.009 |
| BP 🡪 Obstacles on DR-QoL | -0.021 | 2.558 | **.011** | -0.038 | -0.005 |
| **Specific Indirect Effects** |  |  |  |  |  |
| BMI 🡪 FBG 🡪 BP | -0.029 | 2.346 | **.019** | -0.052 | -0.004 |
| BMI 🡪 BP 🡪 Stress | -0.004 | 1.793 | .073 | -0.009 | -0.001 |
| BMI 🡪 FBG 🡪 Stress | 0.184 | 13.906 | **< .001** | 0.158 | 0.210 |
| BMI 🡪 SBP 🡪 Obstacles on DR-QoL | -0.001 | 0.690 | .490 | -0.005 | 0.001 |
| BMI 🡪 FBG 🡪 Obstacles on DR-QoL | 0.126 | 9.685 | **< .001** | 0.100 | 0.151 |
| BMI 🡪 Stress 🡪 Obstacles on DR-QoL | -0.014 | 1.442 | .149 | -0.034 | 0.004 |
| FBG 🡪 BP 🡪 Stress | 0.003 | 1.683 | .092 | 0.000 | 0.009 |
| FBG 🡪 BP 🡪 Obstacles on DR-QoL | 0.001 | 0.682 | .495 | -0.001 | 0.005 |
| FBG 🡪 Stress 🡪 Obstacles on DR-QoL | 0.165 | 13.939 | **< .001** | 0.143 | 0.190 |
| BP 🡪 Stress 🡪 Obstacles on DR-QoL | -0.021 | 2.558 | **.011** | -0.038 | -0.005 |
| BMI 🡪 FBG 🡪 BP 🡪 Stress | 0.002 | 1.670 | .095 | 0.000 | 0.004 |
| BMI 🡪 BP 🡪 Stress 🡪 Obstacles on DR-QoL | -0.001 | 1.779 | .075 | -0.004 | -0.000 |
| BMI 🡪 FBG 🡪 Stress 🡪 Obstacles on DR-QoL | 0.073 | 11.886 | **< .001** | 0.062 | 0.086 |
| BMI 🡪 FBG 🡪 BP 🡪 Obstacles on DR-QoL | 0.000 | 0.681 | .496 | -0.001 | 0.002 |
| FBG 🡪 BP 🡪 Stress 🡪 Obstacles on DR-QoL | 0.001 | 1.676 | .094 | 0.000 | 0.004 |
| BMI 🡪 FBG 🡪 BP 🡪 Stress 🡪 Obstacles on DR-QoL | 0.001 | 1.664 | .096 | 0.000 | 0.002 |
| **Total Effects** |  |  |  |  |  |
| BMI 🡪 Obstacles on DR-QoL | 0.156 | 6.416 | **< .001** | 0.110 | 0.204 |
| BMI 🡪 FBG | 0.442 | 22.231 | **< .001** | 0.403 | 0.481 |
| BMI 🡪 Stress | 0.146 | 6.164 | **< .001** | 0.099 | 0.193 |
| BMI 🡪 BP | 0.041 | 1.658 | .097 | -0.008 | 0.089 |
| FBG 🡪 Obstacles on DR-QoL | 0.451 | 18.452 | **< .001** | 0.401 | 0.496 |
| FBG 🡪 Stress | 0.419 | 17.533 | **< .001** | 0.372 | 0.465 |
| FBG 🡪 BP | -0.065 | 2.365 | **.018** | -0.116 | -0.009 |
| Stress 🡪 Obstacles on DR-QoL | 0.397 | 19.106 | **< .001** | 0.356 | 0.437 |
| BP 🡪 Obstacles on DR-QoL | -0.036 | 1.580 | .114 | -0.082 | 0.008 |
| BP 🡪 Stress | -0.053 | 2.574 | **.010** | -0.094 | -0.013 |

Model was adjusted by age, education, occupation, ethnicity, area, gender, duration of DM, placed diagnose, family history, health insurance, adherence of medication, and drugs. Bold indicates significancy.

BMI, body mass index; CI, confident interval; CIBC, confidence intervals bias corrected; DBP, diastolic blood pressure; DM, diabetes mellitus; DR-QoL, diabetes-related quality of life*;* FBG, fasting blood glucose; *p*, *p* value; SBP, systolic blood pressure.
